# Increased Conformational Flexibility of a Macrocycle–Receptor Complex Contributes to Reduced Dissociation Rates

**DOI:** 10.1002/chem.201702776

**Published:** 2017-08-30

**Authors:** Adrian Glas, Eike‐Christian Wamhoff, Dennis M. Krüger, Christoph Rademacher, Tom N. Grossmann

**Affiliations:** ^1^ Chemical Genomics Centre of the Max Planck Society Otto-Hahn-Str. 15 44227 Dortmund Germany; ^2^ Max Planck Institute of Colloids and Interfaces Department of Biomolecular Systems Am Mühlenberg 1 14424 Potsdam Germany; ^3^ Freie Universität Berlin Department of Biology, Chemistry and Pharmacy Takustr. 3 14195 Berlin Germany; ^4^ VU University Amsterdam Department of Chemistry and Pharmaceutical Sciences De Boelelaan 1083 1081 HV Amsterdam The Netherlands; ^5^ Present address: Uppsala University Department of Cell and Molecular Biology, BMC Box 596 75124 Uppsala Sweden

**Keywords:** ^19^F NMR spectroscopy, binding kinetics, cyclic peptides, molecular dynamics simulation, peptidomimetics

## Abstract

Constraining a peptide in its bioactive conformation by macrocyclization represents a powerful strategy to design modulators of challenging biomolecular targets. This holds particularly true for the development of inhibitors of protein‐protein interactions which often involve interfaces lacking defined binding pockets. Such flat surfaces are demanding targets for traditional small molecules rendering macrocyclic peptides promising scaffolds for novel therapeutics. However, the contribution of peptide dynamics to binding kinetics is barely understood which impedes the design process. Herein, we report unexpected trends in the binding kinetics of two closely related macrocyclic peptides that bind their receptor protein with high affinity. Isothermal titration calorimetry, ^19^F NMR experiments and molecular dynamics simulations reveal that increased conformational flexibility of the macrocycle–receptor complex reduces dissociation rates and contributes to complex stability. This observation has impact on macrocycle design strategies that have so far mainly focused on the stabilization of bioactive ligand conformations.

Many therapeutically relevant bio‐macromolecules cannot be targeted with traditional small molecule approaches.^1^ Often these targets are characterized by relatively shallow surfaces as they are frequently found at the interface of protein–protein interactions (PPI).^2^ Due to their excellent surface recognition properties, large macrocyclic molecules are considered promising candidates to target protein areas involved in PPIs.^3^ This holds particularly true for peptide‐derived macrocycles which display favorable binding properties being linked to their large surface areas as well as their unique structural and dynamic characteristics.^4^ Despite their constrained structure, they exhibit considerable conformational freedom that allows for efficient sampling of bioactive states.3d, 5 In many cases, the effects of macrocyclization on the thermodynamics of binding are well investigated. Often, favorable entropic profiles resulting from reduced flexibilities of the free ligand translate into increased affinities.3d, 6 However, the influence of macrocyclization on the conformational dynamics of the bound state and the binding kinetics are less understood. Notably, the residence time of a bound ligand (reciprocal of dissociation rate) is of particular interest^7^ as it strongly correlates with therapeutic efficacy.^8^ Herein, we investigate two closely related peptide‐derived macrocyclic ligands (**MC18** and **MC22**) binding the same receptor protein (Figure 1 a) but showing distinct binding characteristics. Using isothermal titration calorimetry (ITC), ^19^F NMR titration experiments and molecular dynamics (MD) simulations, we show that increased conformational flexibility of the macrocycle–receptor complex contributes to reduced dissociation rates and thereby to higher complex stability.

**Figure 1:**
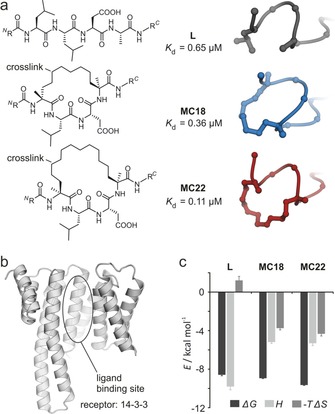
a) Chemical structure of the core region of **L**, **MC18** and **MC22** (^*N*^R=*N*‐trifluoroacetyl glycine‐QG, ^*C*^R=LDLAS) including affinity for 14–3‐3 isoform ζ (derived from ITC) and cartoon representation of their bound conformation (PDB ID 4n7g, 4n7y, 4n84).6a b) Structure of receptor 14‐3‐3 in cartoon representation (PDB ID 4n7y). c) Binding thermodynamics of ligand‐14‐3‐3 complexes (*H*=enthalpy, *S*=entropy, *G*=Gibbs energy, for details see Supplementary methods and Figures S1–S3).

Previously, we reported a set of macrocycles derived from a bacterial peptide binding epitope (**L**, grey) composed of 11 amino acids (Figure 1 a).6a Two macrocyclic peptides show particularly high affinity for the receptor protein 14‐3‐3 isoform ζ (from now on called 14‐3‐3) and were potent inhibitors of its interaction with Exoenzyme S. One of these peptides comprises a macrocycle containing 18 atoms (**MC18**, blue) while the other one bears a 22 atom ring system (**MC22**, red). **MC22** displays the highest affinity for the receptor. The ligand–receptor complexes have been characterized via X‐ray crystallography verifying the central groove of 14‐3‐3 as the binding site for all ligands (Figure 1 b). In addition, it was shown that macrocyclization indeed reduces flexibility in the free state of the ligand. Having access to these well characterized ligand–receptor pairs, we were interested in the impact of the different macrocyclization architectures on binding kinetics. For that purpose, we decided to use ^19^F NMR spectroscopy which provides high sensitivity for the detection of biomolecular interactions.^9^ To enable ligand‐observed NMR experiments, the linear and both macrocyclic peptides were synthesized and N‐terminally modified with *N*‐trifluoroacetyl glycine.

To elucidate binding thermodynamics of the fluorine‐labeled ligands, ITC experiments were performed (Figure 1 c). Compared to linear peptide **L** (dissociation constant, *K*_d_=0.65 μm), both macrocycles exhibit increased affinity for the receptor (**MC18**: *K*_d_=0.36 μm, **MC22**: *K*_d_=0.11 μm). Binding of **L** is enthalpically driven (Δ*H*=−9.81 kcal mol^−1^) with negligible entropic contribution (−*T*Δ*S*=−0.41 kcal mol^−1^). As expected, both macrocyclic ligands show substantially increased entropic contributions (−*T*Δ*S*=−3.75 and −4.34 kcal mol^−1^, for **MC18** and **MC22**, respectively). Interestingly, this is accompanied by reduced binding enthalpy (**MC18**: 1.9‐fold, **MC22**: 1.8‐fold), a trend that is often referred to as enthalpy‐entropy compensation.^10^

Having determined the thermodynamic parameters of all ligand–receptor pairs, we applied ^19^F NMR spectroscopy to investigate the binding kinetics. ^19^F NMR spectra of all peptides alone and in presence of varying receptor concentrations were recorded. Free and bound ligand states are in slow exchange on the ^19^F NMR chemical shift timescale (Figure 2 a) with chemical shift differences (Δ*δ*) of bound and free state resonances ranging between 175 and 598 Hz. In this exchange regime, ^19^F NMR line shape analysis for the free ligands allows for the determination of dissociation rates (*k*_off_, Figure 2 b, Figures S4–S6, Equations S1 and S2).^11^ Notably, 14‐3‐3 proteins form stable homodimers which only exchange very slowly under our conditions.^12^ This is supported by our NMR experiments (Figure S7) and prevents interference with the determination of ligand binding kinetics.^13^

**Figure 2:**
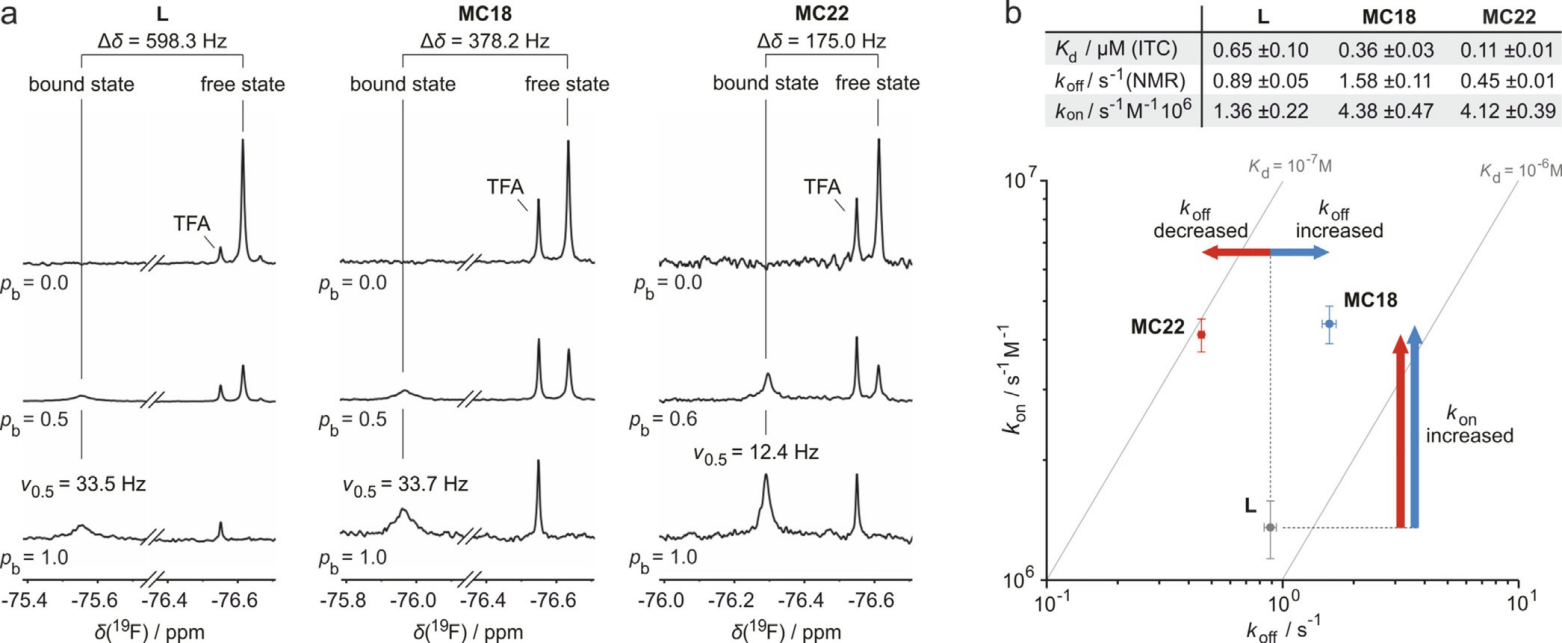
^19^F NMR experiments and binding kinetics. a) ^19^F NMR spectra determined to investigate the interaction between ligands (**L**, **MC18**, **MC22**) and 14‐3‐3. The concentration of 14‐3‐3 increases from top to bottom resulting in an increase of fraction bound (*p*_b_) for the ligands (for details see Supporting Information methods and Figure S4 and S5). b) Binding kinetics for the interaction with 14‐3‐3. Dissociation rates (*k*_off_) were determined via ^19^F NMR line shape analysis (Figure S6). Association rates (*k*_on_=*k*_off_×*K*_d_^−1^) were calculated using *k*_off_‐values and ITC‐derived dissociation constants (*K*_d_). Errors of *K*_d_ (ITC) account for the standard deviation 1σ (triplicate of measurements). Errors of *k*_off_ (NMR) originate from the fitting procedure (Equation S1).

Based on the *k*_off_ values and ITC‐derived dissociation constants, association rates (*k*_on_=*k*_off_×*K*_d_^−1^) were calculated. Compared to **L** (*k*_on_=1.36×10^6^ s^−1^ M^−1^), both macrocyclic peptides exhibit increased association rates (**MC22**: *k*_on_=4.12×10^6^ s^−1^ M^−1^, **MC18**: *k*_on_=4.38×10^6^ s^−1^ M^−1^) clearly indicating a beneficial effect of conformational constraint on the association process. Strikingly, the dissociation rates of both macrocycles differ considerably (Figure 2 b). While **MC18** (*k*_off_=1.58 s^−1^) exhibits accelerated dissociation compared to **L** (*k*_off_=0.89 s^−1^), **MC22** (*k*_off_=0.45 s^−1^) displays a lower dissociation rate. Since both cyclic peptides show similar association rates, the superior affinity of **MC22** is a result of its lower dissociation rate. Considering the closely related structures of **MC18** and **MC22** (Figure 1 a), this is a remarkable observation.

The dissociation process is generally considered to be dominated by the properties of the ligand–receptor complex.^8^, ^14^ The ^19^F NMR spectra provide information about the flexibility of the bound ligand since decreases in linewidth (*ν*_0.5_) correlate with increased flexibility (Figure 2 a).^15^ Interestingly, this analysis indicates higher flexibility for the bound state of macrocycle **MC22** (*ν*_0.5_=12.4 Hz) compared to **MC18** (*ν*_0.5_=33.7 Hz) and linear ligand **L** (*ν*_0.5_=33.5 Hz). While this provides an initial insight into the dynamic properties of these ligand–receptor complexes, it is not clear if this is merely a local effect observed for the fluorine label.

To dissect the different contributions to the properties of the bound state, we employed MD simulations for all three ligand–receptor complexes.^16^ The corresponding crystal structures (PDB ID 4n7g, 4n7y, 4n84) served as a starting point for these calculations. Ligands were prepared using Maestro^17^ and considered as they had been used in ITC and ^19^F NMR experiments (including their N‐terminal trifluoroacetyl glycine). MD simulations were performed for 1.5 μs per complex taking snapshots every 20 ps. Snapshots were clustered using a 2 Å cutoff for the minimum distance between clusters. The cluster probability distributions served to calculate conformational entropies (*S*_conf_) which represent a measure for the conformational flexibility of peptides and proteins.^18^ Comparing conformational entropies of these ligand–receptor complexes with their dissociation rates reveals a clear correlation (Figure 3 a) indicating that increased flexibility of the complex is linked with reduced *k_off_* values. A closer look at the conformational entropies reveals that the complex of **MC22** (*S*_conf_=7.5 kcal mol^−1^) shows increased flexibility compared to the complex of **L** and **MC18** (*S*_conf_=6.6 and 6.4 kcal mol^−1^, Figure 3 b) which is consistent with the above mentioned narrower NMR linewidth (ν_0.5_) for the **MC22**–receptor complex. Interestingly, differences in the overall flexibility appear to originate mainly from the ligands (Figure 3 b).

**Figure 3:**
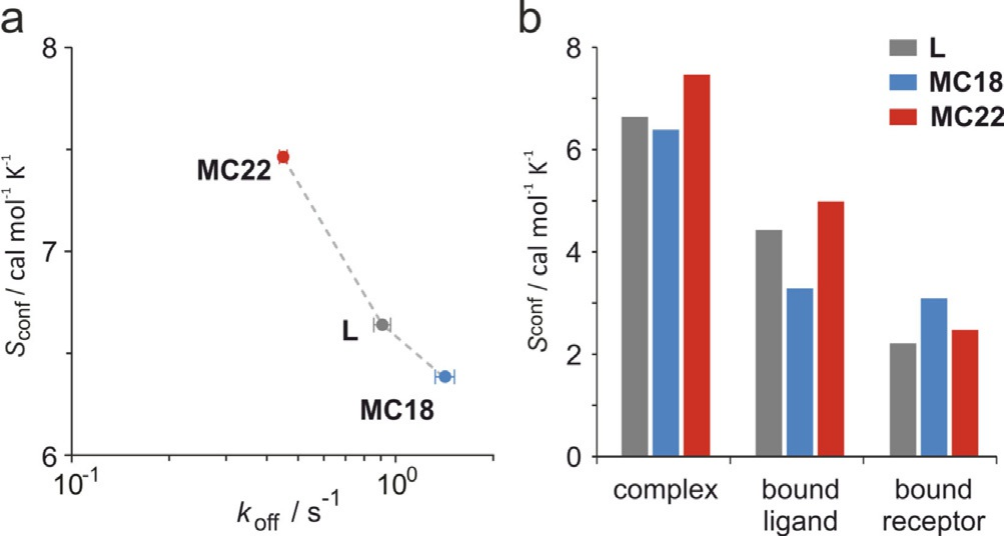
MD‐derived conformational entropies. a) Plot of conformational entropies (*S*_conf_) for ligand–receptor complexes versus dissociation rates (*k*_off_, derived from ^19^F NMR). b) Total conformational entropies of ligand–receptor complexes and individual contributions by ligand and receptor.

Considering the relatively high structural similarity of **MC18** and **MC22**, we were interested to locate the regions responsible for the different flexibilities in the two receptor‐bound macrocycles. For that purpose, the root mean square fluctuations (RMSF) were calculated for all main chain atoms and for the carbon atoms within the ligand crosslink in **MC18** (blue) and **MC22** (red, Figure 4 a, b). Based on these RMSF values, the two macrocycles display similar flexibilities within the peptide core sequence (X^1^LDX^2^, Figure 4 a, X=crosslinking amino acids), but differ in their terminal regions and the crosslink itself (Figure 4 a, b). Here, **MC22** shows considerably higher flexibility than **MC18** mainly contributing to overall differences in conformational entropies of the bound state. A closer look at both bound macrocycles including a color coding for RMSF values illustrates these differences in flexibility (Figure 4 c, d, indicating low (white) to high (orange) flexibility). Both peptides exhibit highest flexibility for their very terminal amino acids which is in line with previously reported crystal structures showing less defined electron density in these regions (PDB ID: 4N7Y and 4N84).6a Notably, both termini in **MC18** exhibit lower flexibility than corresponding areas in **MC22**. In addition, the crosslink in **MC18** appears to be considerably more rigid than in **MC22**. This behavior can be explained by the observation that the crosslink in **MC18** reaches further into the hydrophobic groove of 14‐3‐3 (Figure S10 and S11) which may constrain its conformational freedom. Importantly, these MD findings are in line with the decreased NMR linewidth for the N‐terminal fluorine label in **MC22** (Figure 2 a).

**Figure 4:**
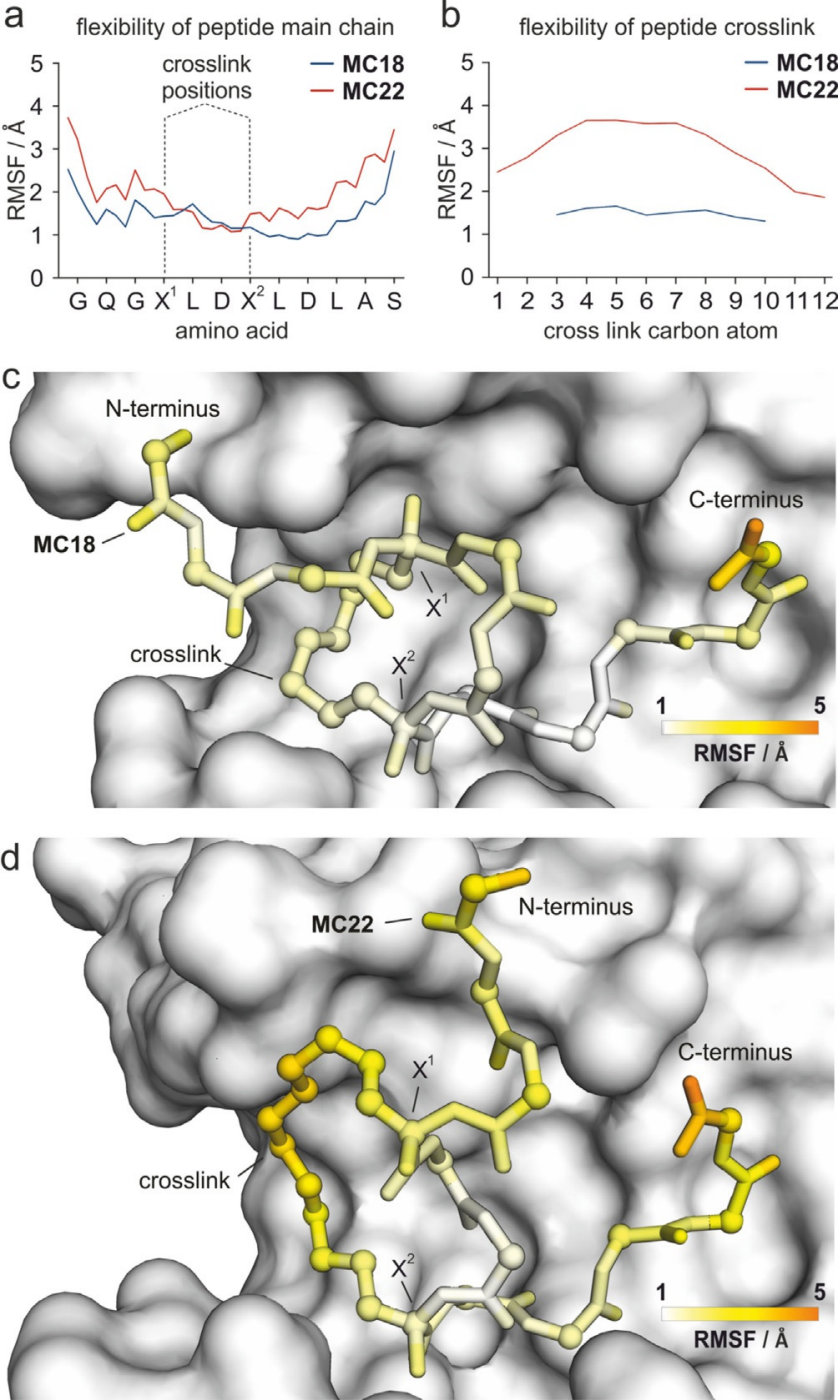
MD‐derived flexibilities. a) RMSF values of peptide main chain atoms (blue: **MC18**; red: **MC22**) in complex with 14–3‐3 (X=modified amino acid for crosslink incorporation). b) RMSF values of crosslink atoms for the bound macrocycles (**MC18** blue; **MC22** red). c, d) Visualization of RMSF‐values (correlating with flexibility) of **MC18** and **MC22** when bound to the receptor. Peptide backbone and crosslink are shown in stick representation with α‐carbons and crosslink carbons highlighted as spheres. Receptor (grey) is shown in surface representation.

Taken together, these results provide mechanistic insight into the contribution of peptide flexibility to receptor binding using ITC and ^19^F NMR experiments combined with MD simulations. Although both macrocycles exhibit similar thermodynamic profiles, ^19^F NMR reveals intriguing differences in binding kinetics. Strikingly, reduced dissociation rates (and thereby increased affinity) correlate with increased conformational flexibilities of the ligand–receptor complex. This observation has implications for the design of high affinity peptides and macrocycles which so far focused on the stabilization of a bioactive conformation in the free state. Our findings suggest complementing this strategy with a consideration of the bound state aiming for increased flexibility. However, we cannot conclude general design principles based on these initial findings. Taken into consideration that in some cases crosslink incorporation was also reported to result in a loss of entropic contributions to binding,^19^ any endeavor towards affinity maturation of macrocyclic ligands is highly advised to involve a thorough biophysical characterization of receptor binding. Even though, such integrated optimization strategies are time and resource demanding, they may provide the possibility to obtain ligands with both increased affinity and prolonged residence time, of which the latter is an important pharmacological parameter towards high drug efficacy. In addition, our findings highlight the potential of loop‐like peptide epitopes as starting point for macrocyclic ligands as they exhibit reduced intramolecular hydrogen bond stabilization when compared to repetitive secondary elements such as α‐helix and β‐sheet. Importantly, loop‐like epitopes are underrepresented in current stabilization approaches that predominantly focus on α‐helices.

## Conflict of interest

The authors declare no conflict of interest.

## Supporting information

As a service to our authors and readers, this journal provides supporting information supplied by the authors. Such materials are peer reviewed and may be re‐organized for online delivery, but are not copy‐edited or typeset. Technical support issues arising from supporting information (other than missing files) should be addressed to the authors.

Supplementary

## References

[1] L. G. Milroy , T. N. Grossmann , S. Hennig , L. Brunsveld , C. Ottmann , Chem. Rev. 2014, 114, 4695–4748. 24735440 10.1021/cr400698c

[2] I. S. Moreira , P. A. Fernandes , M. J. Ramos , Proteins 2007, 68, 803–812. 17546660 10.1002/prot.21396

[3a] T. A. Cardote , A. Ciulli , ChemMedChem 2016, 11, 787–794; 26563831 10.1002/cmdc.201500450 PMC4848765

[3b] P. G. Dougherty , Z. Qian , D. Pei , Biochem. J. 2017, 474, 1109–1125; 28298556 10.1042/BCJ20160619 PMC6511976

[3c] T. A. Hill , N. E. Shepherd , F. Diness , D. P. Fairlie , Angew. Chem. Int. Ed. 2014, 53, 13020–13041; 10.1002/anie.201401058 25287434

[3d] L. Nevola , E. Giralt , Chem. Commun. 2015, 51, 3302–3315. 10.1039/c4cc08565e 25578807

[4a] B. C. Doak , B. Over , F. Giordanetto , J. Kihlberg , Chem. Biol. 2014, 21, 1115–1142; 25237858 10.1016/j.chembiol.2014.08.013

[4b] M. Pelay-Gimeno , A. Glas , O. Koch , T. N. Grossmann , Angew. Chem. Int. Ed. 2015, 54, 8896–8927; 10.1002/anie.201412070 PMC4557054 26119925

[4c] E. A. Villar , D. Beglov , S. Chennamadhavuni , J. A. Porco Jr , D. Kozakov , S. Vajda , A. Whitty , Nat. Chem. Biol. 2014, 10, 723–731. 25038790 10.1038/nchembio.1584 PMC4417626

[5a] H. N. Hoang , K. Song , T. A. Hill , D. R. Derksen , D. J. Edmonds , W. M. Kok , C. Limberakis , S. Liras , P. M. Loria , V. Mascitti , A. M. Mathiowetz , J. M. Mitchell , D. W. Piotrowski , D. A. Price , R. V. Stanton , J. Y. Suen , J. M. Withka , D. A. Griffith , D. P. Fairlie , J. Med. Chem. 2015, 58, 4080–4085; 25839426 10.1021/acs.jmedchem.5b00166

[5b] B. Over , P. Matsson , C. Tyrchan , P. Artursson , B. C. Doak , M. A. Foley , C. Hilgendorf , S. E. Johnston , M. D. T. Lee , R. J. Lewis , P. McCarren , G. Muncipinto , U. Norinder , M. W. Perry , J. R. Duvall , J. Kihlberg , Nat. Chem. Biol. 2016, 12, 1065–1074. 27748751 10.1038/nchembio.2203

[6a] A. Glas , D. Bier , G. Hahne , C. Rademacher , C. Ottmann , T. N. Grossmann , Angew. Chem. Int. Ed. 2014, 53, 2489–2493; 10.1002/anie.201310082 24504455

[6b] H. L. Tran , K. W. Lexa , O. Julien , T. S. Young , C. T. Walsh , M. P. Jacobson , J. A. Wells , J. Am. Chem. Soc. 2017, 139, 2541–2544; 28170244 10.1021/jacs.6b10792 PMC5345905

[6c] R. A. Turner , A. G. Oliver , R. S. Lokey , Org. Lett. 2007, 9, 5011–5014. 17956112 10.1021/ol702228u

[7] J. A. Miles , D. J. Yeo , P. Rowell , S. Rodriguez-Marin , C. M. Pask , S. L. Warriner , T. A. Edwards , A. J. Wilson , Chem. Sci. 2016, 7, 3694–3702. 28970875 10.1039/c5sc04048e PMC5618334

[8a] R. A. Copeland , Nat. Rev. Drug Discovery 2016, 15, 87–95; 26678621 10.1038/nrd.2015.18

[8b] P. J. Tummino , R. A. Copeland , Biochemistry 2008, 47, 5481–5492. 18412369 10.1021/bi8002023

[9a] C. Dalvit , Prog. Nucl. Magn. Reson. Spectrosc. 2007, 51, 243–271;

[9b] N. G. Sharaf , A. M. Gronenborn , Methods Enzymol. 2015, 565, 67–95. 26577728 10.1016/bs.mie.2015.05.014

[10a] J. D. Chodera , D. L. Mobley , Annu. Rev. Biophys. 2013, 42, 121–142; 23654303 10.1146/annurev-biophys-083012-130318 PMC4124006

[10b] J. D. Dunitz , Chem. Biol. 1995, 2, 709–712; 9383477 10.1016/1074-5521(95)90097-7

[10c] C. Forrey , J. F. Douglas , M. K. Gilson , Soft Matter 2012, 8, 6385–6392. 22707976 10.1039/C2SM25160D PMC3374587

[11a] M. V. Krishna Sastry , M. J. Swamy , A. Surolia , J. Biol. Chem. 1988, 263, 14826–14831; 3170566

[11b] G. C. K. Roberts , L. Lu-Yun , Protein NMR Spectroscopy: Practical Techniques and Applications, Wiley, Chichester, 2011;

[11c] T. J. Swift , R. E. Connick , J. Chem. Phys. 1962, 37, 307–320.

[12] M. B. Yaffe , FEBS Lett. 2002, 513, 53–57. 11911880 10.1016/s0014-5793(01)03288-4

[13] E. L. Kovrigin , J. Biomol. NMR 2012, 53, 257–270. 22610542 10.1007/s10858-012-9636-3

[14a] R. A. Copeland , D. L. Pompliano , T. D. Meek , Nat. Rev. Drug Discovery 2006, 5, 730–739; 16888652 10.1038/nrd2082

[14b] A. C. Pan , D. W. Borhani , R. O. Dror , D. E. Shaw , Drug Discovery Today 2013, 18, 667–673. 23454741 10.1016/j.drudis.2013.02.007

[15] A. D. Bain , Prog. Nucl. Magn. Reson. Spectrosc. 2003, 43, 63–103.

[16] H. X. Zhou , M. K. Gilson , Chem. Rev. 2009, 109, 4092–4107. 19588959 10.1021/cr800551w PMC3329805

[17] In *Maestro*, version 9.7, Schrödinger, LLC, New York, **2014**.

[18a] N. Bhattacharjee , P. Biswas , Protein Eng. Des. Sel. 2012, 25, 73–79; 22184455 10.1093/protein/gzr059

[18b] A.-S. Yang , B. Honig , J. Mol. Biol. 1995, 252, 351–365. 7563056 10.1006/jmbi.1995.0502

[19a] A. P. Benfield , M. G. Teresk , H. R. Plake , J. E. DeLorbe , L. E. Millspaugh , S. F. Martin , Angew. Chem. Int. Ed. 2006, 45, 6830–6835; 10.1002/anie.200600844 17001728

[19b] J. P. Davidson , O. Lubman , T. Rose , G. Waksman , S. F. Martin , J. Am. Chem. Soc. 2002, 124, 205–215; 11782172 10.1021/ja011746f

[19c] J. E. Delorbe , J. H. Clements , B. B. Whiddon , S. F. Martin , ACS Med. Chem. Lett. 2010, 1, 448–452; 21116482 10.1021/ml100142y PMC2992351

[19d] D. G. Udugamasooriya , M. R. Spaller , Biopolymers 2008, 89, 653–667. 18335423 10.1002/bip.20983

