# Supplementary material for: Increased Conformational Flexibility of a Macrocycle–Receptor Complex Contributes to Reduced Dissociation Rates
Source: Chemistry. 2017 Aug 30;23(64):16157–61. doi: 10.1002/chem.201702776 (PMC5724689; doi:10.1002/chem.201702776)
Supplement: Supplementary file 1 — Supplementary [file CHEM-23-16157-s001.pdf]

CHEMISTRY
A European Journal

Supporting Information

Increased Conformational Flexibility of a Macrocycle–Receptor Complex Contributes to Reduced Dissociation Rates

Adrian Glas+,[a] Eike-Christian Wamhoff+,[b, c] Dennis M. Krüger,[a, e] Christoph Rademacher,*[b, c] and Tom N. Grossmann*[a, d]

chem_201702776_sm_miscellaneous_information.pdf

Table of Content

1 Methods **2**

1.1 Peptide synthesis and characterization 2

1.2 Protein expression and purification 3

1.3 Isothermal titration calorimetry (ITC) 3

1.4 ^{19}F NMR 4

1.5 Computations 5

2 Supporting tables **7**

2.1 Peptide characterization 7

2.2 Isothermal titration calorimetry (ITC) 8

3 Supporting figures **9**

3.1 Isothermal titration calorimetry (ITC) 9

3.2 ^{19}F NMR 12

4 References **17**

1 Methods

1.1 Peptide synthesis and characterization

1.1.1 General

The peptides were synthesized manually on NovaSyn®TGR Resin (loading 0.24 mmol/g; Merck KGaA) by standard Fmoc-based solid-phase peptide synthesis (SPPS) in polypropylene reaction vessels (Bio-Rad) on a vacuum manifold (Promega).[1] 1 mL solvent was used per 100 mg resin in all reaction and washing steps. For mixing, the resin was purged with nitrogen gas. After each reaction step, the resin was washed 5 times with *N*-methyl-2-pyrrolidinone (NMP), 5 times with dichloromethane (DCM) and 5 times with NMP. Fmoc-protected, proteogenic amino acids were purchased from Iris Biotech. The non-natural olefinic amino acids were purchased from Okeanos Tech. Coupling reagents were purchased at Carl Roth.

1.1.2 Fmoc-deprotection

The resin was swollen in NMP for 15 min and afterwards treated for 15 min with a solution of 25 % piperidine in NMP.

1.1.3 Amino acid coupling

According to the loading of the resin 4 equivalents of the Fmoc-protected amino acids were mixed with 4 equivalents of (Benzotriazol-1-yloxy)tripyrrolidinophosphonium hexafluorophosphate (PyBop) and 8 equivalents of *N,N*-diisopropylethylamine (DIPEA) in NMP and added to the resin for 1 h at room temperature twice. *N*-trifluoroacetyl glycine was coupled according to the procedure of Fmoc-protected amino acids.

1.1.4 *N*-Acetylation (Capping)

After every double coupling, potentially free *N*-terminal amino groups were reacted with NMP/acetic anhydride/DIPEA (10/1/1, v/v/v) for 5 min twice.

1.1.5 Ring closing olefin metathesis (RCM)

The resin was swollen in 1,2-dichloroethane for 10 min in a nitrogen gas flow and afterwards treated with 4 mg mL^{-1} 1st generation Grubbs catalyst 4 times for 2 h. The resin was washed with a mixture of dimethyl sulfoxide (DMSO) and DCM (1/1, v/v) for 10 min.

1.1.6 Reduction

The double bond in the hydrocarbon crosslink which is formed during RCM was reduced using 0.6 M 2,4,6-triisopropylbenzenesulfonyl hydrazide and 1.2 M piperidine (200 µL per 50 mg resin). The resin was treated with the solution 3 times for 100 min at 60 °C and 1000 rpm orbital shaking using a ThermoMixer from Eppendorf.

1.1.7 Peptide cleavage from the resin

Before cleavage, the resin was dried under reduced pressure. The dry resin was treated with a solution of trifluroacetic acid/1,2-ethanedithiol/H_2O/triisopropylsilane (94/2.5/2.5/1, *v*/*v*/*v*/*v*) for 3 h. The Peptides were precipitated with diethyl ether at -20 °C.

1.1.8 Peptide purification and analysis

The peptides were dissolved in H_2O/acetonitrile (1/1, *v*/*v*) and purified by reversed-phase HPLC using a Nucleodur C18 reverse-phase column (10 x 125 mm, 110 Å, particle size 5 µm, Macherey-Nagel; solvent A: water + 0.1 % TFA; solvent B: acetonitrile + 0.1 % TFA; flow rate: 6 mL min^{-1}). Obtained peptides were lyophilized. The purity and the identity of the peptides was investigated by an Agilent HPLC/ESI system equipped with a Zorbax C18 reverse-phase column (4.6 x 150 mm, particle size 5 µm, Agilent; solvent A: water + 0.1% TFA; solvent B: acetonitrile + 0.1% TFA; flow rate: 1 mL min^{-1}). The Data is shown in Table S1. The Peptides were quantified by weight or by comparative HPLC at λ = 210 nM.

1.2 Protein expression and purification

The expression and purification of 14-3-3ζ (aa 1–245) was performed as described before.[2] For ITC and NMR experiments, 14-3-3ζ was used including the His_6-tag.

1.3 Isothermal titration calorimetry (ITC)

ITC experiments were performed at 30°C with a VP-ITC instrument from MicroCal. The sample cell was loaded with 15 µM 14-3-3ζ (aa 1-245) with remaining His-Tag in ITC buffer (10 mM HEPES, 150 mM NaCl, 0.5 mM tris(2-carboxyethyl)phosphine, pH 7.4). Peptides were dissolved in the same buffer (**L** = 190 µM; **MC18** = 200 µM; **MC22** = 150 µM) and injected in 8 µL aliquots with a total number of 35 injections. The duration time between two injections was 180 s. The heating power was monitored. Based on heating power, values for *N*, *K*, Δ*H* and Δ*S* were calculated using Origin 7 (OriginLab Corporation).

1.4 ^{19}F NMR

^{19}F NMR experiments were conducted on a PremiumCompact 600 MHz spectrometer equipped with a OneNMR probe (Agilent). Titration experiments were conducted in 50 mM HEPES, 100 mM NaCl, 2 mM $MgCl_2$, 1% DMSO and 10% D_2O at pH 7.4 and room temperature. Residual TFA from revered-phase HPLC purification was used as an internal reference. Spectra were recorded for samples prepared independently on different days. For **L**, spectra were recorded at a concentration of 11.1 µM at six 14-3-3ζ concentrations ranging from 0 µM to 14.1 µM. For **MC18**, spectra were recorded at a concentration of 11.7 µM at six 14-3-3ζ concentration ranging from 0 µM to 9.2 µM. For **MC22**, spectra were recorded at a concentration of 4.0 µM at six 14-3-3ζ concentration ranging from 0.0 µM to 4.3 µM. The number of scans (*n*) was set to 4096 or 32768, the relaxation delay (d_1) was set to 1.0 s and the acquisition time (t_{acq}) was set to 1.0 s.

Recorded spectra were processed in *MestreNova* (*Version 11.0.2* from *Mestrelab Research* (2016)) via exponential apodization at 0.3 Hz, zero filling with 32k points and an automatic baseline correction utilizing the cubic splines method. Next, processed spectra were exported and ^{19}F NMR line shapes of the resonances corresponding to **L**, **MC18**, **MC22** and TFA were analyzed in *OriginPro* (*Version 9.1* from *OriginLab* (2015)) via fitting a single Lorentzian function to the spectral data points. This analysis served to determine integrals I and linewidths $v_{0.5}$ used during the calculations described below. Notably, for the line shape analysis of **MC22** in presence of 3.6 µM 14-3-3ζ three spectra data points on the right flank of the resonance corresponding to the free state of **MC22** were classified as outliers and not considered during the fitting procedures.

The bound fraction of ligand (p_b) was calculated in a ratiometric approach from the resonances corresponding to free and the bound state of **L**, **MC18** or **MC22** in a single spectrum and served to determine the ratio of bound and free fraction of ligand $p_b*p_f^{-1}$. Linewidths ($v_{0.5}$) of the resonance corresponding to the free state of **L**, **MC18** or **MC22** were utilized to determine dissociation rates (k_{off})[3] for binding equilibria in the slow exchange regime (Equation 1).[4] $R_{2,f}$ corresponds to the transversal relaxation rate of the free state of a ligand in absence of 14-3-3ζ.

$$R_{2,obs} = \pi v_{0.5,obs} = k_{off} p_b p_f^{-1} + R_{2,f}$$

Equation S1

Linewidths ($v_{0.5}$) were determined from a single experiment for each concentration and were corrected for field inhomogeneities via the TFA resonance (Equation 2).[5] Standard errors for k_{off} were estimated directly from the fitting procedure.

$$R_{2,obs} - R_{2,f} = \left(v_{0.5,obs} - v_{0.5,obs}^{TFA}\right) - \left(v_{0.5,f} - v_{0.5,f}^{TFA}\right)$$

Equation S2

1.5 Computations

1.5.1 Structure preparation

The basis of the MD simulations was provided by the X-ray structures of the complexes between the **L** (PDB ID 4N7G), **MC18** (PDB ID 4N7Y) and **MC22** (PDB ID 4N84) peptides and the human adaptor protein 14-3-3ζ, respectively.[2] From each complex, we selected one peptide and the 14-3-3 dimer for MD simulations. The complex structures for MD were prepared using Maestro by adding missing atoms or amino acids.[6] The *N*-terminus of each peptide was capped with *N*-trifluoracetyl glycine.

1.5.2 Molecular dynamics simulation

All MD simulations were conducted with AMBER16 on GPU using Sander for minimization and equilibration, and PMEMD for the production runs.[7] In total, four series of MD simulations in explicit water were performed each with three independent 500ns replicas (in total 1.5 µs runtime), one for the unbound 14-3-3ζ protein, and three for the protein–peptides complexes, resulting in an overall simulation time of 6 µs. Force field parameters for the peptides were taken from the general AMBER force field (GAFF).[8] Atomic charges for the peptides were determined by the AM1-BCC method with SQM and the Amber tool Antechamber.[9] Protein atoms and bound ions were described by the ff14SB force field.[10] All structures were generated with LEaP. Each structure was placed in an octahedral TIP3P water box with an extension of at least 11 Å in each direction from the solute and neutralized by adding Na^+ or Cl^- counter ions.[11] Solvated systems were subjected to a two-step minimization procedure to remove clashes between the water molecules and the solute: (1) 50 steps of steepest descent and 200 steps of conjugate gradient minimization with harmonic positional restraints of strength 25 kcal mol^{-1} A^{-2} on all solute atoms, (2) 50 steps of steepest descent and 200 steps of conjugate gradient minimization with harmonic positional restraints of strength 5 mol^{-1} A^{-2} on all solute atoms. After minimization, four steps of equilibration were run: (1) 50 ps NVT simulation to increase the thermostat target temperature from 100 K to 300 K using Berendsen's temperature and pressure control algorithms with time constants of 0.5 ps for both heat bath coupling and pressure relaxation, (2) 50 ps NPT simulation at constant isotropic pressure of 1 atm to adjust the density of the system to 1 g cm^{-3}, (3) five 50 ps NVT simulations progressively decreasing the restraints in steps of 1 kcal mol^{-1} A^{-2}, and finally (4) 50 ps NVT simulation without any restraints.[12]

For each system, three independent equilibrations followed by three independent 500ns production simulations were performed (for RMSD plots see Figure S8 and S9). The simulation time step was 2 fs and snapshots were saved every 20 ps. During dynamics the SHAKE algorithm was used to constrain all bonds involving hydrogen atoms.[13] For short-range non-bonded interactions, an 8 Å cutoff radius was used. Long-range electrostatic interactions were treated by the Particle Mesh Ewald method.[14] The temperature was

kept constant at 300 K using Berendsen's weak coupling algorithm.[12] Production simulations were carried out under NVT conditions.

Analysis of the MD trajectories was performed employing CPPTRAJ, and visual inspection of the MD snapshots was performed using PyMol (*The PyMOL Molecular Graphics System, Version 1.8, Schrödinger, LLC*). The first 10 ns of each replica were discarded from the analysis as equilibration time, clustering was performed using the hierarchical agglomerative approach with average linkage and a 2 Å cutoff for the minimum distance between the clusters. All frames were aligned based on the backbone atoms. The distance between the frames was calculated via best-fit coordinate RMSD based on all atoms.

Entropic contributions were approximated by calculating conformational entropies based on cluster probability distributions. In this work, the conformational entropy is calculated in terms of Shannon entropy which may be expressed as

$$S = -\Sigma[Pi \cdot \ln(Pi)]$$

Equation S3

where Pi is the probability of a conformation to be part of the ith cluster.[15]

2 Supporting tables

2.1 Peptide characterization

Table S1. List of Peptides (with C-terminal amide) including their sequence, HPLC gradient, retention time (t_R), molecular formula (MF), calculated molecular masses (m/z) for charged ions ($[M+1H]^{1+}$) and found masses (m/z). Amino acids are given in one-letter code.

Peptide	*N*-Term mod.	Sequence	HPLC Gradient[a]	HPLC t_R / min	MF	Calculated m/z	Found m/z
**L**	CF_3	QGLLDALDLAS	1	12.3	$C_{52}H_{85}F_3N_{14}O_{19}$	1268.3	1267.8
**MC18**	CF_3	QG-4_R-LD-4_S-LDLAS	2	10.1	$C_{57}H_{93}F_3N_{14}O_{19}$	1336.4	1335.8
**MC22**	CF_3	QG-6_S-LD-6_S-LDLAS	3	17.8	$C_{61}H_{101}F_3N_{14}O_{19}$	1392.5	1391.9

[a] = gradient 1: 20% B to 70% B in 20 min; gradient 2: 40% B to 70% B in 20 min; gradient 3: 50% B to 70% B in 20 min.

2.2 Isothermal titration calorimetry (ITC)

Table S2. ITC data **L** (* for T = 303.15 K).

	**L** run1	**L** run2	**L** run3	mean value	σ
N	1.20	1.19	1.27	1.22	0.04
K	1.33E+06	1.54E+06	1.82E+06	1.56E+06	2.01E+05
K_d [M]	7.52E-07	6.49E-07	5.49E-07	6.50E-07	8.26E-08
Δ*H* [cal mol^{-1}]	-9959	-10030	-9425	-9805	270
ΔS [cal mol^{-1} K^{-1}]	-4.8	-4.8	-2.5	-4.0	1.1
*T**Δ*S* [cal mol^{-1}]	1464	1443	743	1217	335
Δ*G* [cal mol^{-1}]	-8495	-8587	-8682	-8588	77

Table S3. ITC data **MC18** (* for T = 303.15 K).

	**MC18** run1	**MC18** run2	**MC18** run3	mean value	σ
N	0.88	0.86	0.86	0.87	0.01
K	2.54E+06	2.95E+06	2.79E+06	2.76E+06	1.69E+05
K_d [M]	3.94E-07	3.39E-07	3.58E-07	3.62E-07	2.26E-08
Δ*H* [cal mol^{-1}]	-5349	-5153	-5070	-5191	116.97
ΔS [cal mol^{-1} K^{-1}]	11.7	12.6	12.8	12.4	0.5
*T**Δ*S* [cal mol^{-1}]	-3547	-3820	-3880	-3749	145
Δ*G* [cal mol^{-1}]	-8896	-8973	-8950	-8940	32

Table S4. ITC data **MC22** (* for T = 303.15 K).

	**MC22** run1	**MC22** ITC2	**MC22** ITC3	mean value	σ
N	0.95	0.88	0.88	0.90	0.03
K	8.62E+06	9.79E+06	8.69E+06	9.03E+06	5.36E+05
K_d [M]	1.16E-07	1.02E-07	1.15E-07	1.11E-07	6.33E-09
Δ*H* [cal mol^{-1}]	-5039	-5413	-5484	-5312	195
ΔS [cal mol^{-1} K^{-1}]	15.1	14.1	13.7	14.3	0.6
*T**Δ*S* [cal mol^{-1}]	-4578	-4274	-4153	-4335	178
Δ*G* [cal mol^{-1}]	-9617	-9687	-9637	-9647	30

3 Supporting figures

3.1 Isothermal titration calorimetry (ITC)

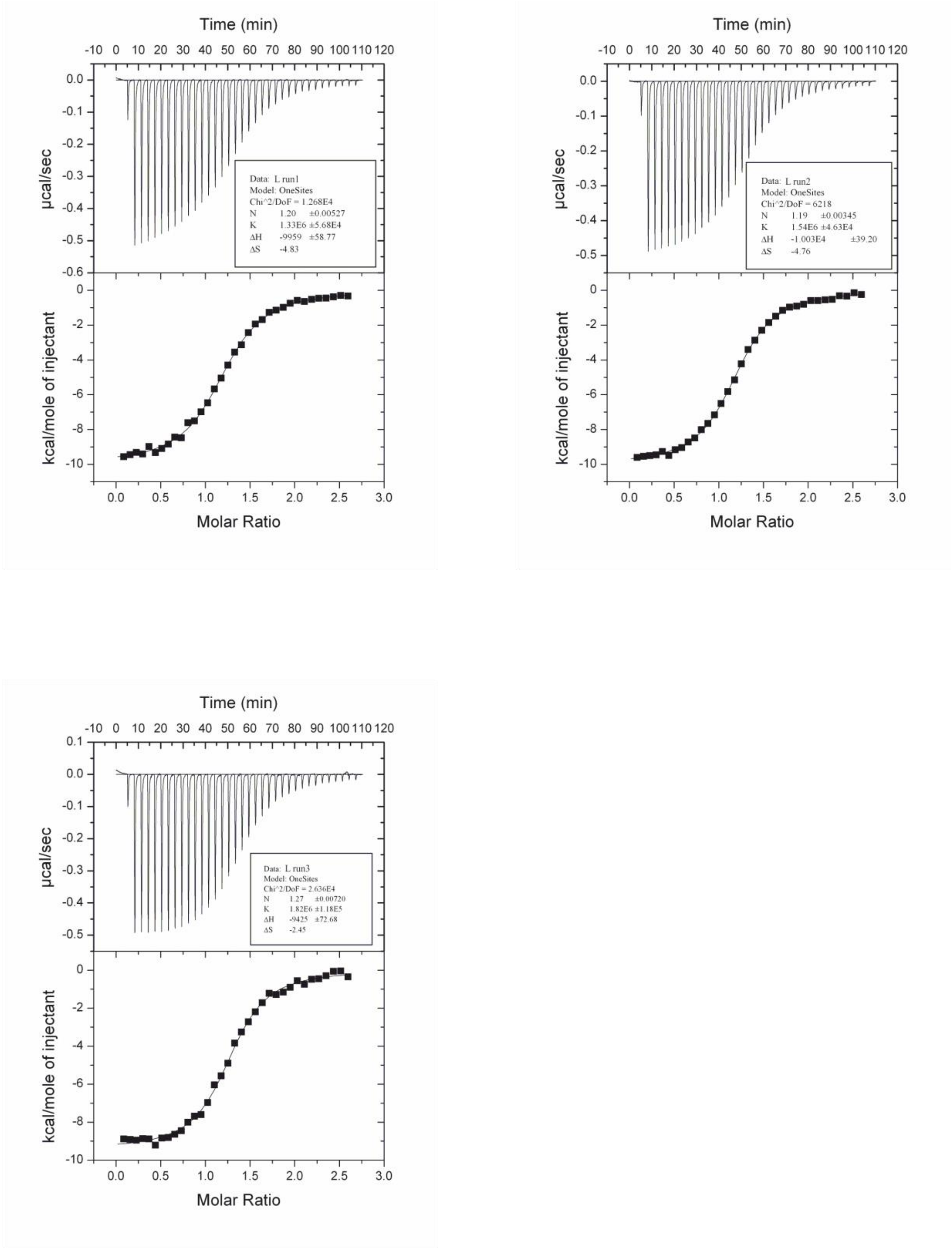

Figure S1. Isothermal Titration Calorimetry (ITC) experiments. 245 µM **L** was titrated into 15 µM full length 14-3-3ζ (both in ITC buffer: 10 mM HEPES (pH 7.4)).

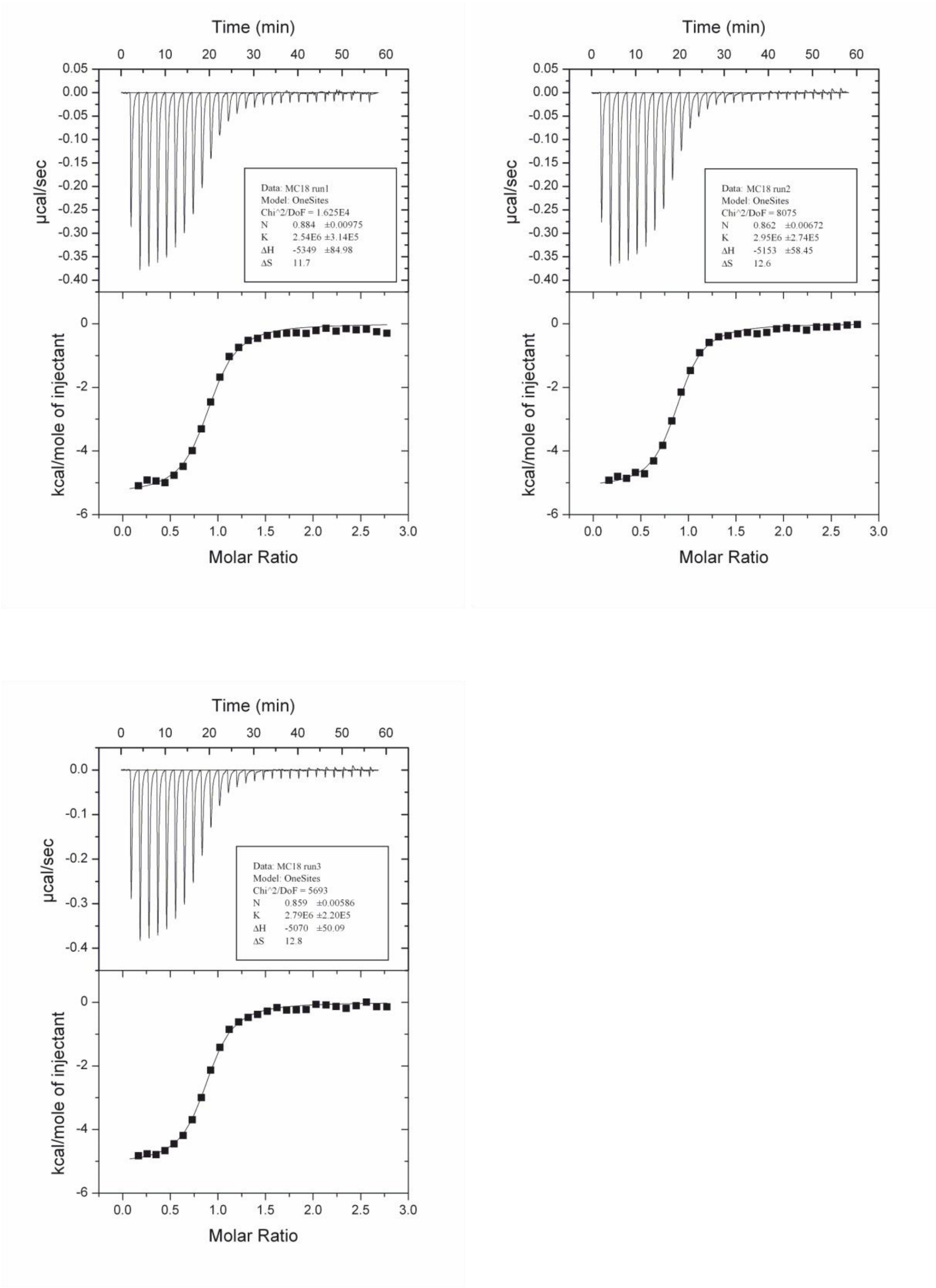

Figure S2. Isothermal Titration Calorimetry (ITC) experiments. 200 µM **MC18** was titrated into 15 µM full length 14-3-3ζ (both in ITC buffer: 10 mM HEPES (pH 7.4)).

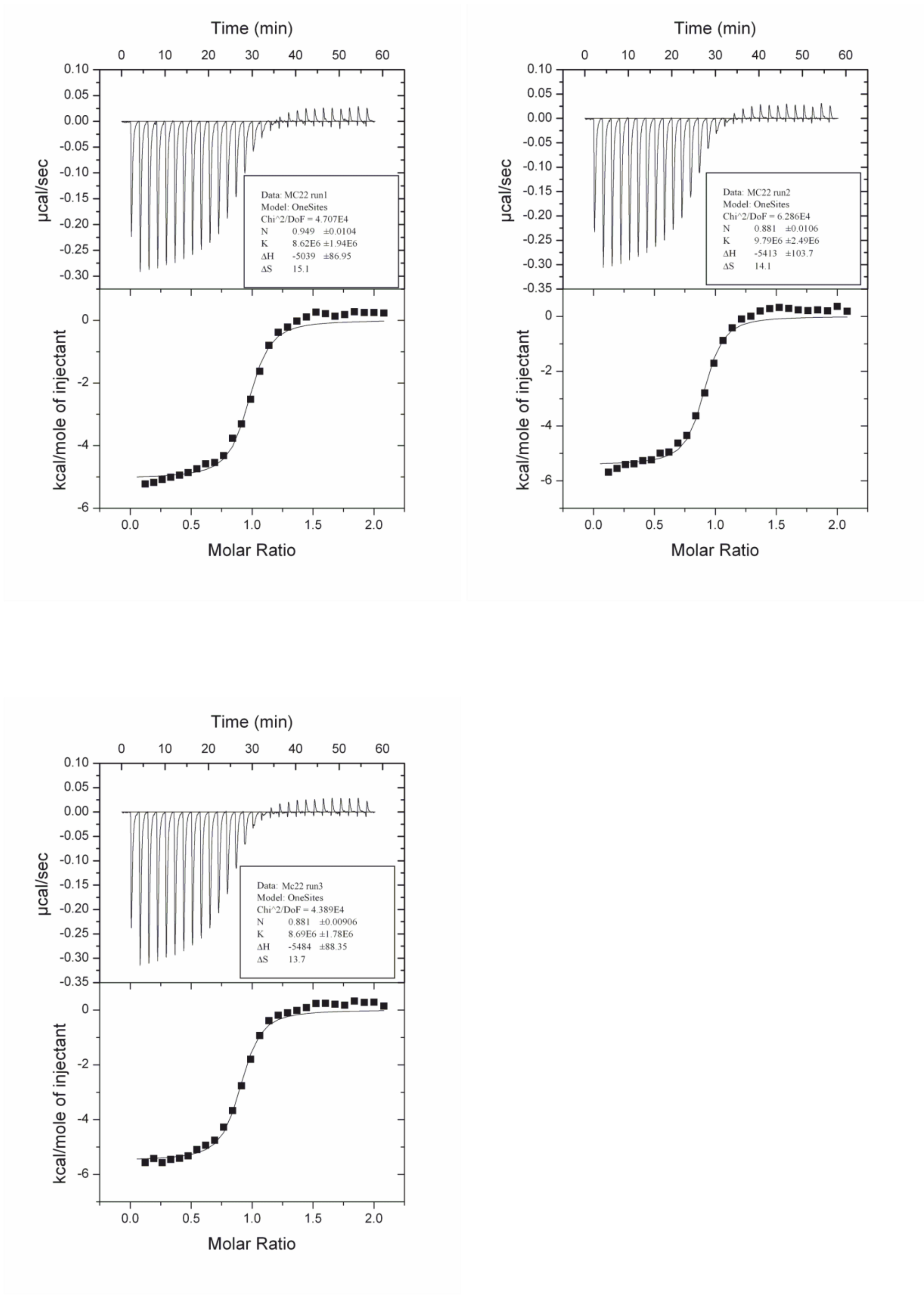

Figure S3. Isothermal Titration Calorimetry (ITC) experiments. 150 µM **MC22** was titrated into 15 µM full length 14-3-3ζ (both in ITC buffer: 10 mM HEPES (pH 7.4)).

3.2 ^{19}F NMR

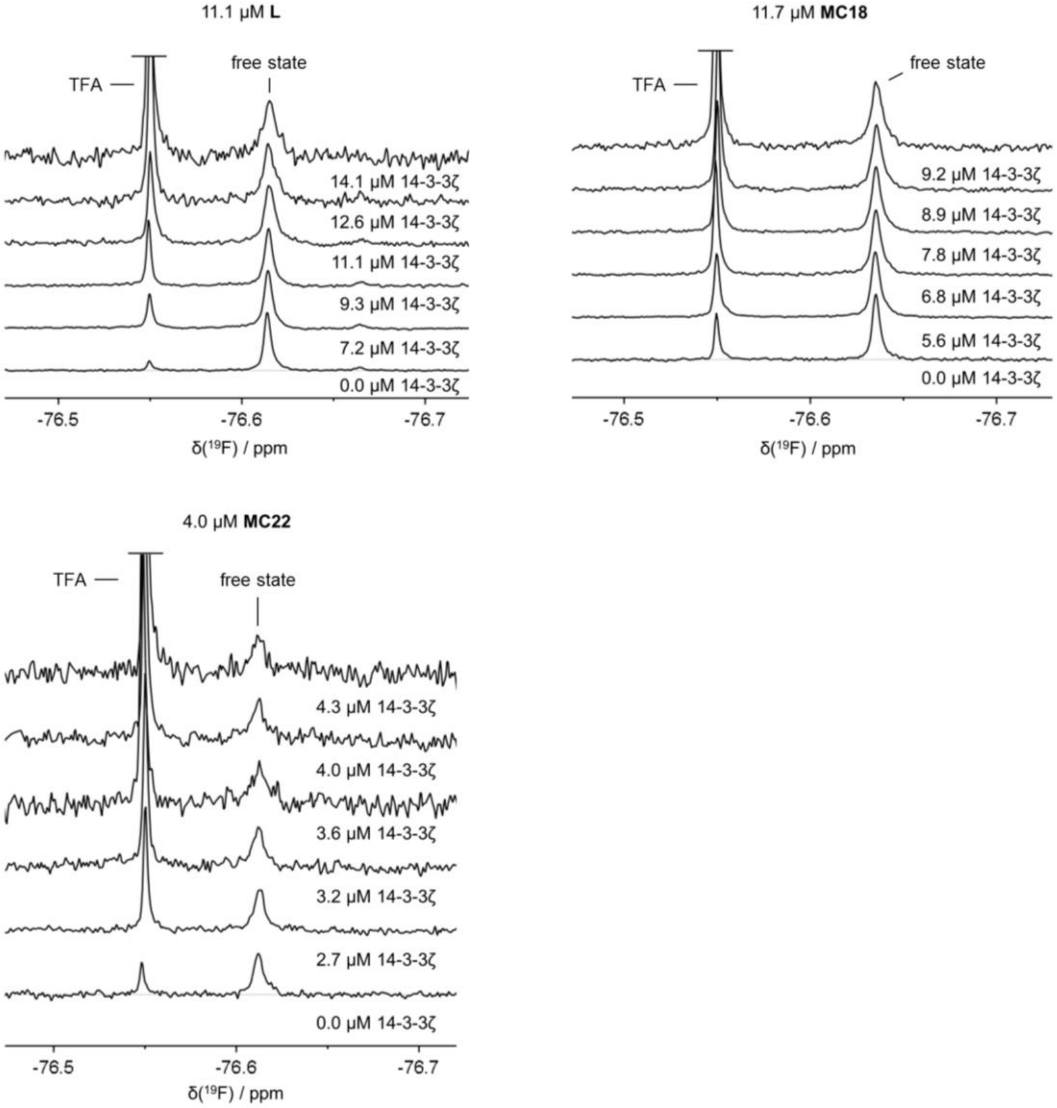

Figure S4, Addition to Figure 2 in manuscript. Titration experiments for ^{19}F NMR line shape analysis. For each spectrum, samples were prepared independently on a different day. Titration experiments were conducted in 50 mM HEPEs, 100 mM NaCl, 2 mM $MgCl_2$, 1% DMSO and 10% D_2O at pH 7.4 and room temperature. For each peptide, the resonances corresponding to residual TFA and the free state are displayed.

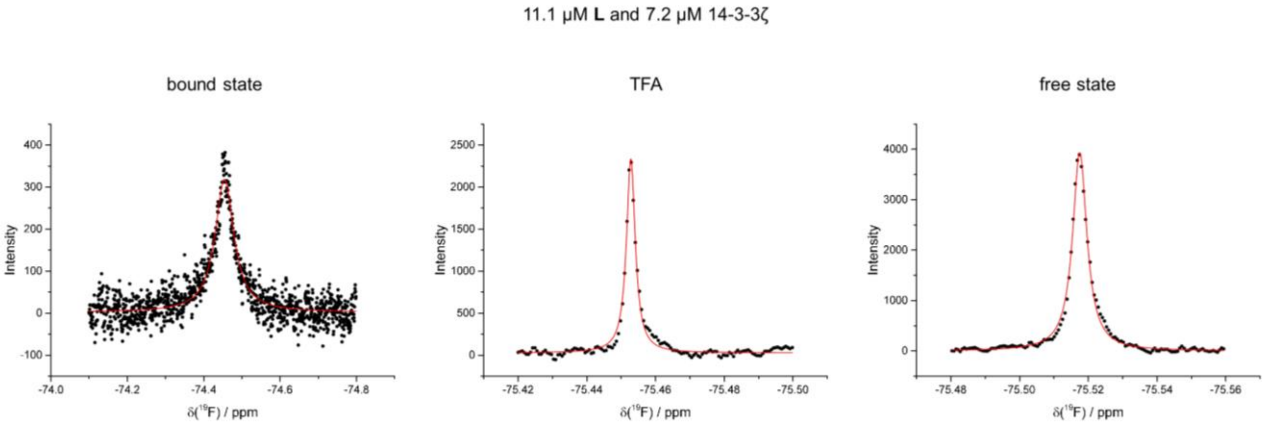

Model	Lorentz		
Equation	y = y0 + (2*I/π)*(v0.5/(4*(x-xc)^2 + v0.5^2))		
Reduced Chi-Sqr	861.7955		
Adj. R-Square	0.855371		
		Value	Standard Error
	y0 / -	0.851291	1.14364
	xc / ppm	-74.4552	4.44E-04
	v0.5 / ppm	0.059286	0.001448
	I	29.52796	0.583983
	H	317.0728	4.754627

Model	Lorentz		
Equation	y = y0 + (2*I/π)*(v0.5/(4*(x-xc)^2 + v0.5^2))		
Reduced Chi-Sqr	2461.119		
Adj. R-Square	0.983062		
		Value	Standard Error
	y0 / -	19.38084	4.87698
	xc / ppm	-75.4529	2.34E-05
	v0.5 / ppm	0.003048	7.01E-05
	I	11.07516	0.192213
	H	2313.007	35.12622

Model	Lorentz		
Equation	y = y0 + (2*I/π)*(v0.5/(4*(x-xc)^2 + v0.5^2))		
Reduced Chi-Sqr	5147.355		
Adj. R-Square	0.991994		
		Value	Standard Error
	y0 / -	-13.7268	7.694193
	xc / ppm	-75.5176	2.54E-05
	v0.5 / ppm	0.005028	7.99E-05
	I	31.10994	0.387391
	H	3939.166	39.59657

Figure S5, Addition to Figure 2 in manuscript. Representative fitting procedures for ^{19}F NMR line shape analysis in OriginPro. The displayed data point corresponds to 11.1 µM L and 7.2 µM 14-3-3ζ in 50 mM HEPEs, 100 mM NaCl, 2 mM $MgCl_2$, 1% DMSO and 10% D_2O at pH 7.4 and room temperature. A single Lorentzian function was fitted to the spectral data points to obtain the integral I and linewidth $v_{0.5}$. Displayed chemical shifts were not referenced to TFA. Notably, the quality of this fitting procedure for the bound state of the ligands did not differ at varying 14-3-3ζ concentrations, excluding a mixed contribution to the line shape from monomer and dimer populations. Furthermore, at all analyzed 14-3-3ζ concentrations, only a single resonance corresponding to the bound sate of **L**, **MC18** or **MC22** was observed.

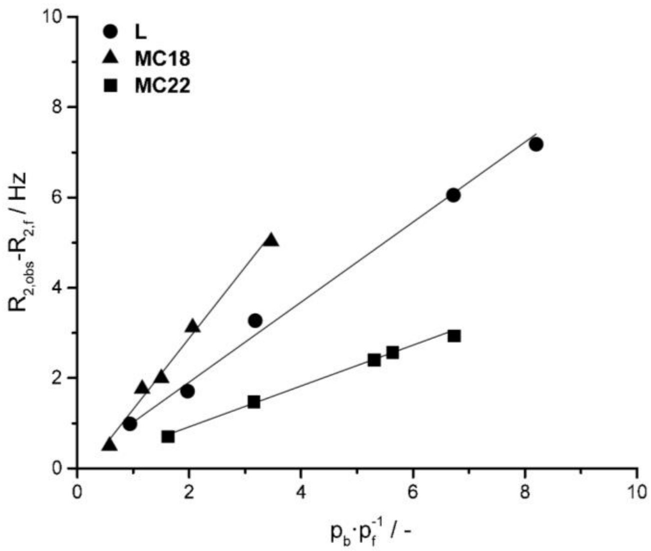

Figure S6, Addition to Figure 2 in manuscript. Determination of k_{off} via ^{19}F NMR line shape analysis. $R_{2,obs}$ – $R_{2,f}$ values were calculated from determined linewidths $v_{0.5,obs}$ of the free state via Equation 2 and plotted against the ratio of bound and free fraction of ligand $p_b{*}p_f^{-1}$. k_{off} values were determined via fitting Equation 1 to the plotted data points. For all three ligands a linear relationship is maintained over the analyzed range of $p_b{*}p_f^{-1}$ values corresponding to varying 14-3-3ζ concentrations. This observation is consistent with the formation of kinetically stable dimers by 14-3-3ζ at micromolar concentration. Under these conditions, the monomer-dimer equilibrium does not interfere with the determination of k_{off} values.[16] While observed for other ligand–14-3-3ζ systems, the ^{19}F NMR line shape analysis provides no evidence for allosteric cooperativity.[17]

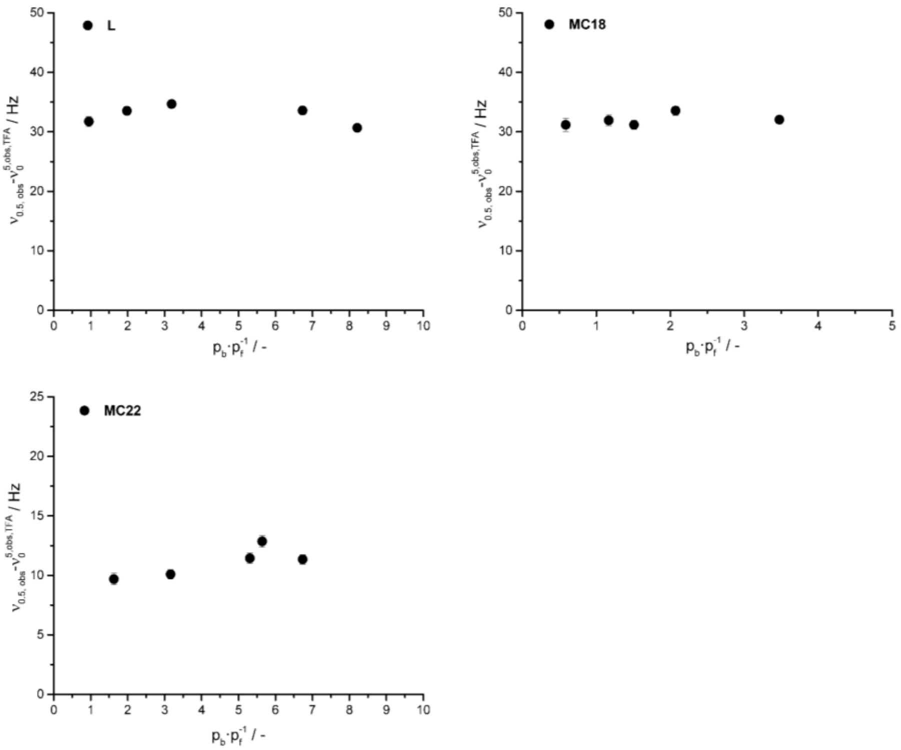

Figure S7. Linewidths of the bound state of the **L**, **MC18** and **MC22**. Upon titration with 14-3-3ζ, no systematic increase of the linewidth $\nu_{0.5}$ is observed for the resonance corresponding to the bound state of either ligand. This observation is consistent with the formation of kinetically stable dimer by 14-3-3ζ at micromolar concentration. Under these conditions, the monomer-dimer equilibrium does not interfere with the ^{19}F NMR line shape analysis.[16]

3.3 Computations

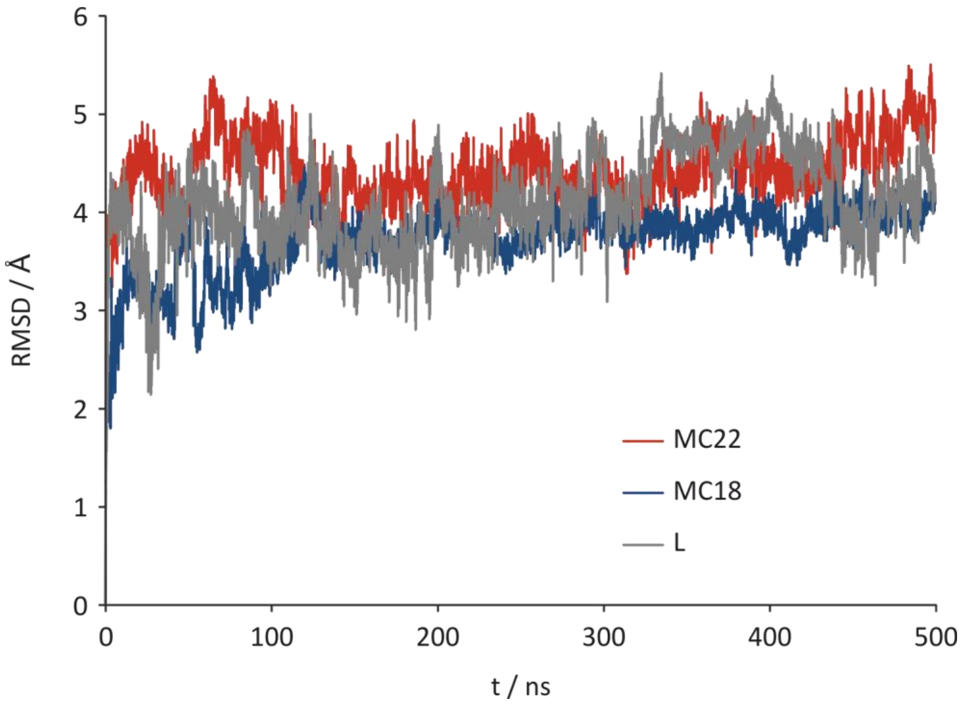

Figure S8: RMSD values of peptide backbone atoms relative to starting structure (peptides bound to 14-3-3).

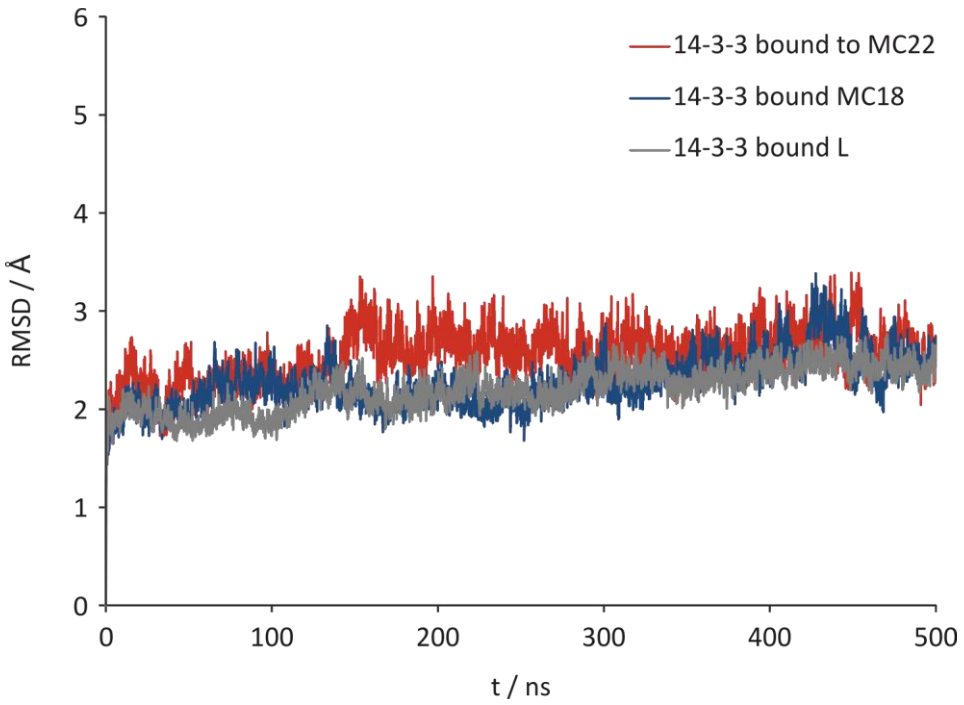

Figure S9: RMSD values of 14-3-3 backbone atoms relative to starting structure (14-3-3 bound to corresponding peptide).

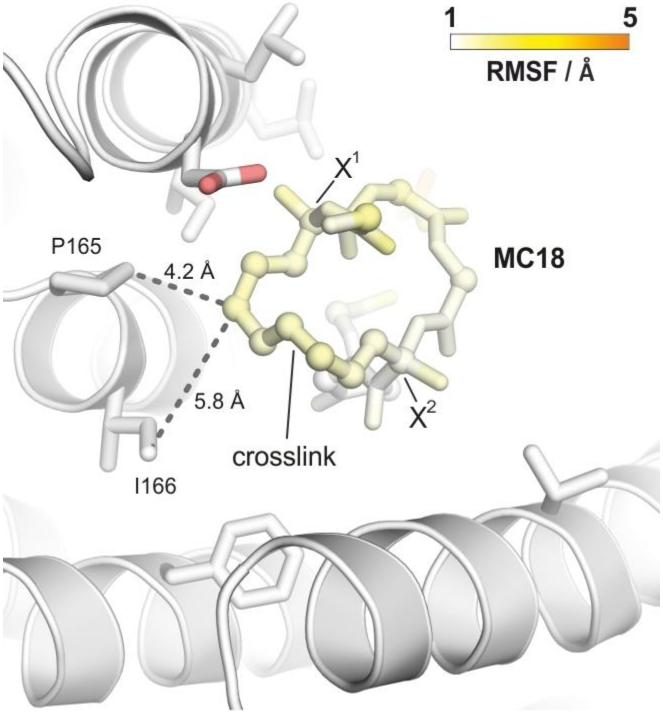

Figure S10: MD-derived structure of the **MC18**–14-3-3 complex. **MC18** backbone and crosslink are shown in stick representation with α-carbons and crosslink carbons highlighted as spheres. Atoms are colored in accordance to their RMSF-value. 14-3-3 protein (grey) is shown as cartoon with selected side chains in stick representation.

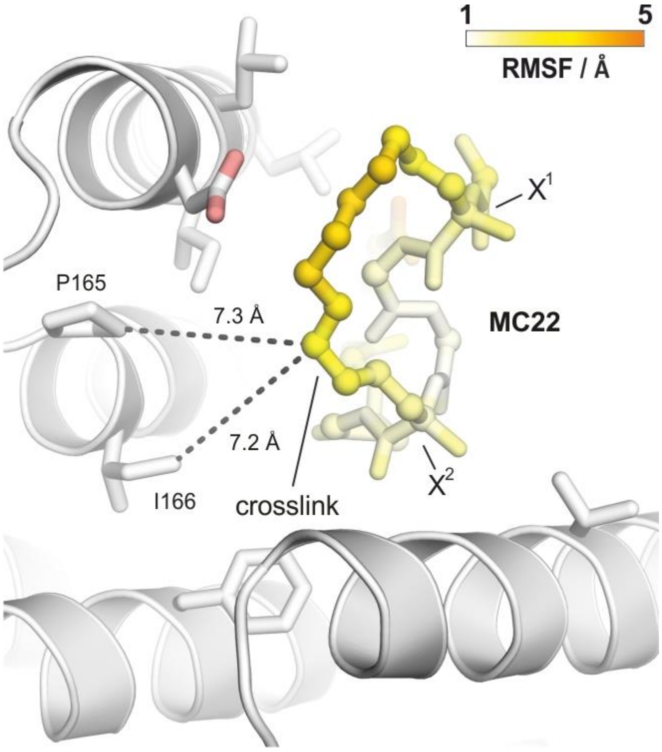

Figure S11: MD-derived structure of the **MC22**–14-3-3 complex. **MC22** backbone and crosslink are shown in stick representation with α-carbons and crosslink carbons highlighted as spheres. Atoms are colored in accordance to their RMSF-value. 14-3-3 protein (grey) is shown as cartoon with selected side chains in stick representation.

4 References

[1] Y. W. Kim, T. N. Grossmann and G. L. Verdine, *Nat. Protoc.* **2011**, *6*, 761-771.

[2] A. Glas, D. Bier, G. Hahne, C. Rademacher, C. Ottmann and T. N. Grossmann, *Angew. Chem. Int. Ed.* **2014**, *53*, 2489-2493.

[3] a) G. C. K. Roberts and L. Lu-Yun, *Protein NMR spectroscopy: practical techniques and applications*, **2011**, p; b) M. V. Krishna Sastry, M. J. Swamy and A. Surolia, *J. Biol. Chem.* **1988**, *263*, 14826-14831.

[4] T. J. Swift and R. E. Connick, *J. Chem. Phys.* **1962**, *37*, 307-320.

[5] N. K. Sauter, M. D. Bednarski, B. A. Wurzburg, J. E. Hanson, G. M. Whitesides, J. J. Skehel and D. C. Wiley, *Biochemistry* **1989**, *28*, 8388-8396.

[6] in *Maestro, version 9.7*, *Vol.* Schrödinger, LLC, New York, **2014**.

[7] a) J. T. B. D.A. Case, R.M. Betz, D.S. Cerutti, T.E. Cheatham, III, T.A. Darden, R.E. Duke, T.J. Giese, H. Gohlke, A.W. Goetz, N. Homeyer, S. Izadi, P. Janowski, J. Kaus, A. Kovalenko, T.S. Lee, S. LeGrand, P. Li, T. Luchko, R. Luo, B. Madej, K.M. Merz, D. M. York and P. A. Kollman in *AMBER 2015*, *Vol.* University of California, San Francisco, **2015**; b) A. W. Gotz, M. J. Williamson, D. Xu, D. Poole, S. Le Grand and R. C. Walker, *J. Chem. Theory. Comput.* **2012**, *8*, 1542-1555; c) R. Salomon-Ferrer, A. W. Gotz, D. Poole, S. Le Grand and R. C. Walker, *J. Chem. Theory. Comput.* **2013**, *9*, 3878-3888.

[8] J. M. Wang, R. M. Wolf, J. W. Caldwell, P. A. Kollman and D. A. Case, *J. Comput. Chem.* **2004**, *25*, 1157-1174.

[9] a) A. Jakalian, B. L. Bush, D. B. Jack and C. I. Bayly, *J. Comput. Chem.* **2000**, *21*, 132-146; b) A. Jakalian, D. B. Jack and C. I. Bayly, *J. Comput. Chem.* **2002**, *23*, 1623-1641; c) J. M. Wang, W. Wang, P. A. Kollman and D. A. Case, *J. Mol. Graph. Model.* **2006**, *25*, 247-260.

[10] a) V. Hornak, R. Abel, A. Okur, B. Strockbine, A. Roitberg and C. Simmerling, *Proteins: Struct. Funct. Bioinf.* **2006**, *65*, 712-725; b) J. A. Maier, C. Martinez, K. Kasavajhala, L. Wickstrom, K. E. Hauser and C. Simmerling, *J. Chem. Theory. Comput.* **2015**, *11*, 3696-3713.

[11] W. L. Jorgensen, J. Chandrasekhar, J. D. Madura, R. W. Impey and M. L. Klein, *J. Chem. Phys.* **1983**, *79*, 926-935.

[12] H. J. C. Berendsen, J. P. M. Postma, W. F. Vangunsteren, A. Dinola and J. R. Haak, *J. Chem. Phys.* **1984**, *81*, 3684-3690.

[13] J. P. Ryckaert, G. Ciccotti and H. J. C. Berendsen, *J. Comput. Phys.* **1977**, *23*, 327-341.

[14] a) T. Darden, D. York and L. Pedersen, *J. Chem. Phys.* **1993**, *98*, 10089-10092; b) U. Essmann, L. Perera, M. L. Berkowitz, T. Darden, H. Lee and L. G. Pedersen, *J. Chem. Phys.* **1995**, *103*, 8577-8593.

[15] a) N. Bhattacharjee and P. Biswas, *Protein Eng. Des. Sel.* **2012**, *25*, 73-79; b) A.-S. Yang and B. Honig, *J. Mol. Biol.* **1995**, *252*, 351-365.

[16] E. L. Kovrigin, *J. Biomol. NMR* **2012**, *53*, 257-270.

[17] J. Hritz, I. J. Byeon, T. Krzysiak, A. Martinez, V. Sklenar and A. M. Gronenborn, *Biophys. J.* **2014**, *107*, 2185-2194.
